# Quantitative Analysis of Ginger Maturity and Pulsed Electric Field Thresholds: Effects on Microstructure and Juice’s Nutritional Profile

**DOI:** 10.3390/foods14152637

**Published:** 2025-07-28

**Authors:** Zhong Han, Pan He, Yu-Huan Geng, Muhammad Faisal Manzoor, Xin-An Zeng, Suqlain Hassan, Muhammad Talha Afraz

**Affiliations:** 1 School of Food Science and Engineering, South China University of Technology, Guangzhou 510641, China; panh20020128@163.com (P.H.); yhgeng@scut.edu.cn (Y.-H.G.); suqlainsolgi@gmail.com (S.H.); 2 Guangdong Provincial Key Laboratory of Intelligent Food Manufacturing, Foshan University, Foshan 528225, China; faisaluos26@gmail.com (M.F.M.); xazeng@scut.edu.cn (X.-A.Z.); 3 Overseas Expertise Introduction Center for Discipline Innovation of Food Nutrition and Human Health (111 Center), Guangzhou 510641, China

**Keywords:** pulsed electric field, ginger maturity, nutritional profile, juice yield, cell disintegration

## Abstract

This study used fresh (young) and old (mature) ginger tissues as model systems to investigate how plant maturity modulates the response to pulsed electric field (PEF), a non-thermal processing technology. Specifically, the influence of tissue maturity on dielectric behavior and its downstream effect on juice yield and bioactive compound extraction was systematically evaluated. At 2.5 kV/cm, old ginger exhibited a pronounced dielectric breakdown effect due to enhanced electrolyte content and cell wall lignification, resulting in a higher degree of cell disintegration (0.65) compared with fresh ginger (0.44). This translated into a significantly improved juice yield of 90.85% for old ginger, surpassing the 84.16% limit observed in fresh ginger. HPLC analysis revealed that the extraction efficiency of 6-gingerol and 6-shogaol increased from 1739.16 to 2233.60 µg/g and 310.31 to 339.63 µg/g, respectively, in old ginger after PEF treatment, while fresh ginger showed increases from 1257.88 to 1824.05 µg/g and 166.43 to 213.52 µg/g, respectively. Total phenolic content (TPC) and total flavonoid content (TFC) also increased in both tissues, with OG-2.5 reaching 789.57 µg GAE/mL and 336.49 µg RE/mL, compared with 738.19 µg GAE/mL and 329.62 µg RE/mL in FG-2.5. Antioxidant capacity, as measured by ABTS^•+^ and DPPH^•^ inhibition, improved more markedly in OG-2.5 (37.8% and 18.7%, respectively) than in FG-2.5. Moreover, volatile compound concentrations increased by 177.9% in OG-2.5 and 137.0% in FG-2.5 compared with their respective controls, indicating differential aroma intensification and compound transformation. Structural characterization by SEM and FT-IR further corroborated enhanced cellular disruption and biochemical release in mature tissue. Collectively, these results reveal a maturity-dependent mechanism of electro-permeabilization in plant tissues, offering new insights into optimizing non-thermal processing for functional food production.

## 1. Introduction

Ginger has long been valued both as a food and medicinal ingredient. In recent years, the popularity of ginger products, such as ginger juice, powder, and essential oil, has surged due to their health-promoting properties. Recognized as generally safe by the FDA, ginger is increasingly used as a functional food and dietary supplement [1,2]. Ginger is a rich source of over 60 active compounds, divided into volatile and non-volatile categories, including shogaols, paradols, gingerols, and zingerone [3]. Known for its anti-inflammatory, antioxidant, and antiglycemic properties, ginger has been widely used for therapeutic purposes, including weight management, nausea relief, and the improvement of muscle, joint, and cardiovascular health [4,5,6,7,8,9,10]. The unique flavor and aroma of ginger, attributed to its pungent and antiseptic compounds, make it a key ingredient in various food products such as sauces, cured meats, curry powders, confectionery, and soft drinks [11]. Its use extends to the production of tinctures, essences, oleoresins, and ginger oil, further enhancing its versatility in the food industry [12].

However, traditional processing methods for ginger and ginger juice can negatively impact its quality, due to factors such as mechanical processing and heat exposure. These methods can lead to the degradation of bioactive compounds, flavor, and texture, as well as lower extraction yields of important constituents like gingerol and shogaol [13,14,15]. Given the rising consumer demand for healthier, minimally processed foods, there is a significant push toward innovative, sustainable extraction methods. Pulsed electric field (PEF) is an emerging non-thermal technique that addresses the limitations of conventional processing methods. It has the potential to enhance juice yield, preserve bioactive compounds, and retain flavor and aroma, while at the same time extending shelf life [16]. Fundamentally, PEF employs short, high-voltage electric pulses to induce electroporation—poration of the cell membrane via the buildup of a transmembrane potential (TMP) that exceeds a critical threshold—thereby increasing membrane permeability and facilitating the release of intracellular constituents [17]. As a non-thermal process, PEF preserves heat-sensitive nutrients such as vitamins and antioxidants and maintains the native sensory attributes of fruits and vegetables [18]. Moreover, it deactivates spoilage microorganisms by damaging cellular walls and denatures detrimental enzymes through conformational changes, thus enhancing microbial safety and prolonging product shelf life without thermal degradation [19,20]. In food processing, particularly juice extraction, PEF has been shown to improve extraction efficiency and yield across various matrices, including apples [21], pomelo [22], grapes [23], yam [24], beetroot [25], and tomatoes [26], often resulting in higher concentrations of phenolic and other bioactive compounds when compared with traditional methods [27].

This study aims to elucidate how the maturity of ginger tissue influences its response to pulsed electric field (PEF) treatment, with a focus on improving extraction efficiency and preserving quality attributes in ginger juice. By using fresh (young) and old (mature) ginger as model systems, we systematically compare their dielectric properties and investigate how these properties affect energy absorption, cell disintegration, and subsequent release of bioactive compounds. Specifically, we evaluate the impact of PEF on the microstructure of ginger slices and the physicochemical, phytochemical, and volatile profiles of the resulting juice. Through this comparative approach, we seek to clarify the mechanisms by which tissue maturity regulates the efficiency of non-thermal extraction and the quality retention of ginger juice.

## 2. Materials and Methods

### 2.1. Materials

The following chemicals and reagents were variously purchased from either Aladdin Industrial Corporation or Macklin Biochemical Corporation, both located in Shanghai, China: ethanol, methanol, sodium chloride, acetic acid (HPLC grade), acetonitrile (HPLC grade), sodium hydroxide, sodium carbonate, sodium nitrite, gallic acid standard, rutin standard, quercetin standard, (+)-catechin, vanillin, 1,1-diphenyl-2-picrylhydrazyl (DPPH^•^), 2,2-azino-bis(3-ethylbenzothiazolin-6-sulfonic acid) diammonium salt (ABTS^•+^), ferrous chloride, ferrozine, aluminum chloride, aluminum trichloride, sodium acetate, 2-octanol. Standards of gingerol (HPLC grade) and shogaols (HPLC grade) were obtained from Chroma Biotechnology Co., Ltd., Chengdu, China. Hydrochloric acid was supplied by Guangzhou Chemical Reagent Factory, Guangzhou, China.

### 2.2. Sample Preparation and Treatment Methods

#### 2.2.1. Sample Preparation

Fresh and aged (10–12 months) ginger (Zingiber officinale Roscoe) samples were sourced from a ginger candy manufacturing company in Chaozhou, Guangdong, China. The samples were thoroughly washed, air-dried, sliced into 1–2 mm pieces, and preserved by dipping in liquid nitrogen. They were then stored at −80 °C to maintain their molecular and physical integrity. Efforts were made to standardize the shape and quality of the samples, ensuring consistency in the test data.

#### 2.2.2. PEF Treatment

PEF treatment was performed using a bench-scale system (Guangzhou Xin-An Food Technology Co., Ltd., Guangzhou, China). Various parameters were tested, including treatment durations (50–150 s), frequencies (10–20 Hz), and pulse widths (6–10 μs) on the fresh and aged ginger slices. Unipolar square waveform pulses were applied. A 30 g sample of ginger slices was placed in the treatment cell and filled with distilled water to the 3 cm mark to ensure uniform conductivity, with an initial conductivity of 226 μS/cm.

The optimal conditions from preliminary trials were selected based on the highest observed total phenolic content and cell disintegration index—a treatment duration of 150 s, a frequency of 20 Hz, and a pulse width of 10 μs. To study the effects of different PEF powers/intensities on the ginger’s microstructure and nutritional profile, we selected 1.5, 2.0, 2.5, and 3.0 kV/cm for both fresh and aged ginger samples. The post-treatment temperature remained below 45 °C. After treatment, the ginger slices were ground, placed in a muslin cloth pouch, and mechanically pressed to extract the juice. The PEF-treated ginger juice samples with the different electric field strengths were labeled as FG (control), FG-1.5, FG-2.0, FG-2.5, and FG-3.0 for fresh ginger, and OG (control), OG-1.5, OG-2.0, OG-2.5, and OG-3.0 for aged ginger. The actual treatment time was 30 milliseconds, with the specific energy applied during PEF treatment ranging from 2.02–8.08 kJ (13.5–53.9 kJ/kg), calculated using Equations (1)–(4).

(1) n=f×t

where n is the of pulses, f is frequency (Hz) and t is the treatment time (s).

(2) $$t_{a}=n\times \tau number$$

where $t_{a}$ is actual treatment time (msec) and τ is pulse width.

(3) $$Q_{s}=n\times \sigma \times \tau \times E^{2}$$

where $Q_{s}$ is energy density (J/m^3^), σ is electrical conductivity (S/m), τ is pulse width (sec), and E is PEF strength (V/m).

(4) $$Q=Q_{s}\times V$$

where Q is specific energy applied (kJ) and V is active treatment volume (m^3^).

Membrane disintegration was assessed by measuring the electrical conductivity before and after PEF treatment using a digital conductivity meter (DDS-307A, INSEA Scientific Instrument Co., Ltd., Shanghai, China). Disintegration was calculated by comparing the conductivity of the intact and damaged samples, with complete disruption determined by heating at 95 °C for 20 min. The degree of disintegration was calculated using Equation (5), as described by Wang, et al. [28].

(5) $$Z=\frac{d-di}{dd-di}$$

where Z denotes the membrane disintegration degree, d is the EC after treatments, di is the EC before treatments and dd is EC of the totally destroyed sample (after heat treatment).

### 2.3. Physicochemical Analysis

#### 2.3.1. Juice Yield

The juice yield of the ginger samples following PEF treatment was calculated using Equation (6):

(6) $$Y\left(\%\right)=\frac{m_{2}-m_{1}}{m_{i}}\times 100$$

where $m_{2}$ is the mass of the extracted juice and the tube (g), $m_{1}$ is the mass of the empty tube (g), and $m_{i}$ is the initial mass of ginger slices (g).

#### 2.3.2. Electrical Conductivity (EC), Temperature, pH, °Brix

EC, pH, temperature (°C), and °Brix were measured using a digital conductivity meter (DDS-307A, INSEA Scientific Instrument Co., Ltd., Shanghai, China), a pH meter (PHS-3E, INSEA Scientific Instrument Co., Ltd., China), a thermometer, and a refractometer (PAL-1, ATAGO Co., Ltd., Tokyo, Japan), respectively. Measurements were taken immediately after PEF treatment. The pH meter was calibrated with commercial buffer solutions (pH 7.0 and pH 4.0). Total soluble solids were determined (at 25 ± 1 °C) as °Brix, and the refractometer prism was cleaned with distilled water after each analysis.

#### 2.3.3. Cloud Value, Cloud Stability, and Non-Enzymatic Browning (NEB)

The cloud value of the ginger juice samples was measured following the method adapted from Aadil, et al. [29]. A 5 mL sample was centrifuged at 3000 rpm for 10 min at 4 °C (H1750R, Cence, Hunan, China). The absorbance of the supernatant at 660 nm was measured using a spectrophotometer (UV-1800, Macy Instrument Co., Ltd., Shanghai, China), with distilled water as the blank.

Cloud stability was assessed using the method outlined by Faisal Manzoor, et al. [30]. Two 5 mL samples from each juice were taken, with one set centrifuged at 4700 rpm for 15 min. The supernatant was separated into different tubes, and absorbance was measured at 625 nm for both sets. Cloud stability was calculated using Equation (7).

(7) $$CS\%=\left(\frac{A_{a}}{A_{b}}\right)\times 100$$

where $A_{a}$ and $A_{b}$ are the absorbances after and before centrifugation, respectively.

Non-enzymatic browning (NEB) was determined following Ahmed, et al. [31]. A 5 mL ginger juice sample was centrifuged at 8000 rpm for 10 min. The supernatant was filtered through a 0.45 µm filter, and the NEB value was measured by absorbance at 420 nm.

### 2.4. Phytochemical Analysis

#### 2.4.1. Determination of Total Phenolic Content (TPC)

TPC of the ginger juice was determined using the Folin–Ciocalteu method, as described by Mustafa and Chin [32]. A 100 µL sample of ginger juice was diluted to 1 mL with distilled water. To this solution, 0.5 mL of Folin–Ciocalteu reagent (1:1 diluted with d.H_2_O) and 2.5 mL of 20% (*w*/*v*) sodium carbonate solution were added. The mixture was mixed and left to react for 40 min at room temperature in the dark. Absorbance was measured at 725 nm using a spectrophotometer (UV-1800, Macy Instrument Co., Ltd., Shanghai, China). The phenolic content was expressed as µg gallic acid equivalents (GAEs) per mL of juice.

#### 2.4.2. Determination of Total Flavonoid Content (TFC)

TFC of ginger juice was determined using the aluminum chloride colorimetric method, as outlined by Zhishen, et al. [33]. A 100 µL sample of ginger juice was diluted to 2 mL with distilled water. To this, 0.15 mL of 5% (*w*/*v*) sodium nitrite solution was added and incubated for 6 min. Then, 0.15 mL of 10% (*w*/*v*) aluminum chloride solution was added and incubated for another 6 min at room temperature. Next, 2 mL of 4% (*w*/*v*) sodium hydroxide was added, and the volume was adjusted to 5 mL with distilled water. The mixture was allowed to stand in the dark for 15 min before the absorbance was measured at 510 nm using a spectrophotometer. Flavonoid content was expressed as µg rutin equivalents (REs) per mL of juice.

#### 2.4.3. Determination of Total Flavanols

Total flavanols were determined following Tanweer, et al. [34] with slight modifications. A 100 µL sample of ginger juice was diluted to 1 mL with distilled water, then mixed with 3 mL of 5% sodium acetate solution and 1 mL of 2% aluminum trichloride solution. After 150 min, absorbance was measured at 440 nm using a spectrophotometer. Flavanol content was expressed as µg quercetin equivalents (QEs) per mL of juice. The same chemicals were used for control and blank samples without ginger juice.

#### 2.4.4. Condensed Tannin Contents

The condensed tannin content in the ginger juice was determined using the method described by Bayoï [35]. A 1.5 mL of 4% vanillin solution (in methanol) was added to 100 µL of ginger juice, followed by 750 µL of concentrated hydrochloric acid (HCl). The mixture was incubated for 25 min at room temperature, and the absorbance was measured at 500 nm using a spectrophotometer.

### 2.5. Antioxidant Analysis

#### 2.5.1. Determination of 1,1-Diphenyl-2-picrylhydrazyl (DPPH^•^) Radical Scavenging Activity

The DPPH^•^ radical scavenging ability of the PEF of the treated ginger juice was assessed using the method described by Sowndhararajan, et al. [36]. Ginger extracts at concentrations of 10, 25, 50, 75, and 100 µL/mL were prepared in ethanol. Equal volumes (100 µL) of each concentration were mixed with 0.1 mM DPPH^•^ solution and left in the dark for 20 min at room temperature. DPPH^•^ reduction was measured at 517 nm using a microplate reader (SpectraMax iD3, Molecular Devices Co., Ltd., San Jose, CA, USA). The scavenging activity was calculated using Equation (8).

(8) $${DPPH}^{•}\;inhibition\;\left(\%\right)=\left(1-\frac{A_{1}-{\;A}_{2}}{A_{0}}\right)\times \;100\%$$

where $A_{0}$ is the absorbance of the mixture containing DPPH^•^ and ethanol, $A_{1}$ is the absorbance of the mixture of sample and DPPH^•^ and $A_{2}$ is the absorbance of the mixture of sample and ethanol.

#### 2.5.2. Determination of 2,2′-Azinobis (3-Ethyl-benzothiozoline-6-sulfonic Acid) Radical Cation (ABTS^•+^) Decolorization

The free radical scavenging activity of all treated ginger juice samples was assessed using the ABTS^•+^ radical cation decolorization assay, as described by Re, et al. [37]. A 7 mM ABTS^•+^ solution was prepared by dissolving disodium salt in distilled water. The ABTS^•+^ radical cation was generated by mixing the ABTS^•+^ stock solution with 2.45 mM potassium persulfate and letting it stand in the dark for 12–16 h. Prior to the assay, the solution was diluted with ethanol and equilibrated at 30 °C to achieve an absorbance of 0.70 ± 0.02 at 734 nm. Various concentrations (10, 25, 50, 75, 100 µL/mL) of ginger juice samples were prepared in ethanol. The ABTS^•+^ working solution was added to each concentration at a 1:15 ratio and incubated at room temperature for 30 min in the dark. Absorbance was measured at 734 nm using a microplate reader (SpectraMax iD3, Molecular Devices Co., Ltd., CA, USA). The ABTS^•+^ scavenging activity was calculated using Equation (9).

(9) $${ABTS}^{•+}scavenging\;rate\left(\%\right)=\left(1-\frac{A_{1}-A_{2}}{A_{0}}\right)\times 100\%$$

#### 2.5.3. Metal Chelating Potential

Metal ion chelation activity was determined following Xie, et al. [38] with minor modifications. A 100 µL sample was mixed with 0.9 mL distilled water, followed by 50 µL of 2 mM FeCl_2_ and 100 µL of 5 mM ferrozine solution. The total volume was adjusted to 3 mL with distilled water, and the mixture was left at room temperature for 10 min. Absorbance was measured at 562 nm, with double-distilled water as the control. The chelating effect was calculated using Equation (10).

(10) $$MC\;\left(\%\right)=\frac{A_{blank}-A_{sample}}{A_{blank}}\times \;100$$

where A is the absorbance of the sample solution and the blank.

### 2.6. In Vitro Studies of Ginger

#### 2.6.1. Microstructure Analysis

The microstructure of randomly selected ginger slices was examined using a scanning electron microscope (Hitachi Regulus-8100, Tokyo, Japan) after drying. The analysis was performed at 3 kV with an 8 nm spot size. A small sample of each ginger slice, treated with PEF treatment, was mounted on a conductive tape and sputtered with platinum (Pt) for 240 s at 30 mA under vacuum (Quorum 150RS Plus, Laughton, UK). The working distance was 5 to 10 mm. The microstructure was examined at magnifications of 150× to 1000×, and representative images were captured for further analysis.

#### 2.6.2. Optical Properties

The color of treated ginger juice samples was analyzed using a colorimeter (DS-01, CHNSpec Technology Co., Ltd., Hangzhou, Zhejiang, China) at room temperature (25 °C). Color was measured using the CIE lab model (L*, a*, b*), where L* denotes lightness, a* represents the red/green axis, and b* corresponds to the yellow/blue axis. The samples, placed in glass Petri dishes, were tested in triplicate. Color difference ((∆E), saturation (C*), hue angle ($h^{0}$),), and whitening index (WI) were calculated using Equations (11)–(14).

(11) $$∆E=\sqrt{{\left(L*-{L*}_{0}\right)}^{2}+{\left(a*-{a*}_{0}\right)}^{2}+{\left(b*-{b*}_{0}\right)}^{2}}$$

(12) $$C*=\sqrt{\left(a^{2}-b^{2}\right)}$$

(13) $${Hue(h}^{0})={tan}^{-1}(b*/a*)$$

(14) $$WI=100-\sqrt{\left(100-L*\right)+{a*}^{2}+{b*}^{2}}$$

#### 2.6.3. Fourier Transform Infrared (FT-IR) Spectroscopy

Infrared spectra of PEF treated dried ginger juice samples were recorded using an FT-IR spectrometer with a Spotlight 400 microscope imager (PerkinElmer, Shelton, CT, USA). The sample was placed on the sensor and gently pressed using the rotating knob. Scanning ranged from 4000 to 650 cm^−1^, with 16 scans at a resolution of 4 cm^−1^.

#### 2.6.4. Determination of Gingerols and Shogaols

The ginger juice was analyzed using HPLC with an Agilent 1260 Infinity II LC system (Santa Clara, CA, USA), following methods by Zhong, et al. [39] and Varakumar, et al. [40]. Separation was performed with an Atlantis T3-C18 column (4.6 mm × 250 mm, 5 μm), with a mobile phase of 0.2% acetic acid (solvent A) and acetonitrile (solvent B). The gradient was as follows: 0–1 min at 0–45% B, 1–13 min at 45–65% B, 13–19 min at 65–80% B, and 19–35 min at 80% B. The flow rate was 1.0 mL/min, injection volume 20 µL, detection at 280 nm, and column temperature at 25 °C. Each sample was injected three times and stored in amber bottles at 4 °C to prevent degradation.

#### 2.6.5. Determination of Volatile Compounds by HS-SPME GC-MS

Headspace solid-phase microextraction gas chromatography–mass spectrometry (HS-SPME GC-MS) analysis was performed using an Agilent 8890 gas chromatograph coupled with an Agilent 7000D triple quadrupole mass spectrometer, equipped with an electron impact ionization chamber. Separation occurred on an HP-INNO-wax column (30 m, 250 µm ID, 0.25 µm film thickness). A 50/30-µm PDMS/DVB/CAR-coated (polydimethylsiloxane/Divinylbenzene/Carboxen) SPME fiber was used for volatile compound extraction, after conditioning at 250 °C for 30 min. For HS conditions, 10 mL of the ginger juice was placed in a 20 mL vial with 3 g NaCl, sealed, and equilibrated at 40 °C for 20 min. The fiber was exposed 1 cm above the liquid surface for 20 min with magnetic stirring at 100 rpm. GC-MS analysis followed the procedure of Yu, et al. [41] and Chen, et al. [42], with minor modifications. Component identification was undertaken by comparing the mass spectra with the NIST20 mass spectral library. Only compounds with a similarity score > 85 were reported. Quantitative analysis was conducted by adding 200 µL of 2-octanol (0.4 mg/mL) as an internal standard, and the relative content was determined by comparing the peak areas.

#### 2.6.6. Statistical Analysis

Data were analyzed using one-way ANOVA, and, for significant differences, Duncan’s multiple comparison tests were applied. Statistical analyses were conducted with IBM SPSS Statistics 25 (SPSS Inc., Chicago, IL, USA), and the experimental result images were prepared using Origin2025.

## 3. Results and Discussion

### 3.1. Juice Yield, Temperature, pH, and °Brix %, Electrical Conductivity (EC) of Ginger

Table 1 collates the physicochemical properties of FG and OG after PEF treatment. The temperature of the FG treatment media increased from 24.17 °C to 43.39 °C, while the OG temperature rose from 22.97 °C to 41.99 °C as the PEF strength was increased from 0 to 3.0 kV/cm. This temperature increment is attributed to the direct thermal effect of electrical pulses, which generate heat in the ginger tissues [43]. While both FG and OG experienced a rise in temperature, OG exhibited a slightly lower increase compared with FG, indicating that old ginger may have different thermal conductivity or a distinct response to the applied electrical pulses. The observed temperature difference between FG and OG suggests that factors such as the moisture content or cellular structure may influence how ginger absorbs and dissipates heat. PEF treatment induces localized heating due to tissue resistance to the electrical field, with the heating effect becoming more pronounced at higher PEF strengths. Although PEF is classified as a non-thermal treatment, it generates heat via ohmic heating, which raises the temperature of the treated samples [44]. These results are consistent with the findings of Yan, et al. [45], in which the sample temperature increases during PEF treatment for microorganism inactivation, due to ohmic heating. Similarly, Zderic and Zondervan [46] have reported that PEF treatment elevates temperatures due to electrical damage to cell membranes and ohmic heating.

The pH of the treatment media for FG was initially 7.23 and gradually decreased with increasing PEF strength. At 3.0 kV/cm, the pH dropped to 6.98. For OG, the pH started at 6.51 and followed a similar trend, decreasing to 6.30 at the highest PEF strength (3.0 kV/cm) (Table 1). This trend suggests that PEF treatment may have caused slight acidification of the treatment media, likely due to the breakdown of organic acids or the release of hydrogen ions from damaged cell membranes. OG consistently showed lower pH values compared with FG across all PEF treatments, which could be attributed to inherent differences in the biochemical composition or cell wall characteristics between FG and OG. The acidification observed could result from cell membrane disruption, which releases intracellular components, including organic acids. The pH difference between FG and OG may also reflect age-related biochemical changes. OG might have different enzyme activities or ion concentrations than FG, making it more susceptible to pH changes. Meneses, et al. [47] have stated that external voltage applications cause ion migration, leading to compositional and chemical changes such as pH variations in the fluids. Kayalvizhi, et al. [48] also observed a decline in pH due to PEF treatment.

The electrical conductivity (EC) values of the treatment media for FG increase with higher PEF strengths, ranging from 223.37 µS/cm (control) to 390.33 µS/cm at 3.0 kV/cm. Similarly, EC in the OG treatment media increased from 254.0 µS/cm (control) to 372.7 µS/cm at the highest PEF strength. This increase in EC indicates that PEF treatment facilitated the release of soluble ions from the ginger cells, likely due to the breakdown of cell membranes or leakage of electrolytes. OG exhibited slightly higher EC values at lower PEF strengths compared with FG, but the increase in EC at higher PEF strengths was similar in both. The rise in EC is a typical response to PEF treatment, as the electric field induces a breakdown of cellular structures, releasing ions and other solutes into the surrounding medium. The higher EC values in OG compared with FG at lower PEF strengths suggest that OG may have a higher electrolyte concentration or a more easily damaged cell structure, making it more prone to ion leakage under PEF treatment. Nowacka, et al. [49] also observed an increase in EC following PEF treatment of plant material. It was suggested that this increase may be linked to the permeability of the cell membrane, which facilitates the leakage of materials like mineral salts into the intercellular space, thus enhancing conductivity.

The °Brix value for FG increased from 2.33% (control) to 3.20% at the highest PEF strength, while OG also showed an increase in °Brix from 3.25% (control) to 4.60% at 3.0 kV/cm. This suggests that PEF treatment enhanced the release of soluble solids, likely due to the breakdown of cellular compartments, which facilitated the release of sugars and other soluble compounds. The °Brix for OG was higher compared with FG at all PEF strengths, which may be related to a higher concentration of soluble solids in OG, possibly due to a more mature storage form of sugars or higher initial sugar content. The increase in °Brix indicates that PEF caused the release of soluble compounds, such as sugars and other organic molecules, into the surrounding solution. This can be attributed to the disruption of the cell membrane structure and enhanced permeability, allowing more dissolved solids to leach out. The higher °Brix in OG compared with FG suggests that the aging process may increase the concentration of soluble solids in ginger. Li and Padilla-Zakour [50] also found that PEF treatment caused an increase in °Brix during their study on the evaluation of PEF and high pressure processing (HPP) on the quality of grape juice.

Juice yield increased as PEF strength increased from 1.5 to 2.5 kV/cm, with the highest yield observed at 2.5 kV/cm (84.16%). The yield slightly decreased at 3.0 kV/cm (80.57%), but it remained higher than the control (69.94%). Similarly, old ginger showed an increase in yield with PEF treatment. The highest yield was observed at 2.5 kV/cm (90.85%), with a slight decrease at 3.0 kV/cm (89.19%). The control yield for old ginger was 81.93%. PEF treatment caused an increase in yield for both FG and OG, particularly at 2.5 kV/cm. However, the slight decrease in yield at 3.0 kV/cm observed in both ginger tissues may be attributed to over-softening of the tissue matrix. Excessive electroporation at higher field strengths can lead to structural collapse of the cellular network and obstruction of micro-channels responsible for juice transport [51]. This over-processing effect results in cellular debris and collapsed cells entrapping the juice, thereby reducing pressing efficiency compared with the optimal condition at 2.5 kV/cm. The decrease in yield at 3.0 kV/cm for both fresh ginger and old ginger may suggest that higher PEF strengths could lead to over-activation of cellular processes or excessive damage to the tissue, which might reduce the yield. Schilling, et al. [52] observed that PEF application resulted in a 1.7 to 7.7% increase in apple juice yield. Lamanauskas, et al. [53] also found an increase of 9–25% in juice yield for PEF-treated raspberries in comparison to untreated samples.

### 3.2. Cloud Value and Cloud Stability of Ginger

Cloud value increased slightly in FG from 2.46 (control) to 2.59 at 2.5 kV/cm and then slightly decreased at 3.0 kV/cm (2.57). The highest cloud value in FG was observed at 2.5 kV/cm. Cloud value for OG was higher than FG across all treatments, starting at 2.73 (control) and reaching a peak of 2.86 at 2.5 kV/cm (Table 1). Cloud value is a measure of the suspended particles or cloudiness in the ginger extract, which may be due to the presence of insoluble compounds or colloidal particles. The increase in cloud value at moderate PEF strengths (2.0 to 2.5 kV/cm) for both FG and OG suggests that PEF treatment facilitates the release of cellular components that increase cloudiness. However, the slight decrease in cloud value at 3.0 kV/cm may indicate a threshold beyond which excessive PEF strength leads to over-dissociation of cellular components, possibly resulting in the loss of these particles or their aggregation. Ertugay, et al. [54] have found that the cloud value of apple juice increases due to PEF treatment, as PEF inactivates enzymes responsible for reducing cloudiness through degradation. The results of this study are consistent with the findings of Rahaman, et al. [55], who also observed an increase in the cloud value of apricot juice due to PEF treatment.

Cloud stability for FG increased from 71.98% (control) to 92.38% at 2.5 kV/cm and slightly decreased to 86.68% at 3.0 kV/cm (Table 1). OG exhibited similar trends, with cloud stability improving from 82.83% (control) to 91.96% at 2.5 kV/cm and decreasing to 90.48% at 3 kV/cm. The increase in cloud stability indicated that PEF treatment, particularly at 2.5 kV/cm, improved the colloidal stability of the ginger juice, likely due to the stabilization of suspended particles or reduced aggregation of components contributing to cloudiness. However, the decrease in stability at 3 kV/cm suggested that excessive PEF treatment may destabilize the colloidal system by over-dissociating particles or disrupting interactions that maintain the cloud stability. Our findings align with those of Wibowo, et al. [56], who applied a 12.5 kV/cm electric field to cloudy apple juice both before and after 3 weeks of storage at 4 °C, demonstrating that PEF treatment effectively maintains cloud stability. However, Timmermans, et al. [57] applied a 23 kV/cm electric field to orange juice and observed that PEF treatment results in the greatest reduction in cloud stability compared with mild heat treatment and high-pressure processing (HPP) during 115 days of storage at 4 °C. The degree of cloud stability achieved primarily depends on the intended application of the product, whether high or low cloud stability is required.

### 3.3. Total Phenolic and Flavonoid Content of Ginger Juice

**Figure 1:**
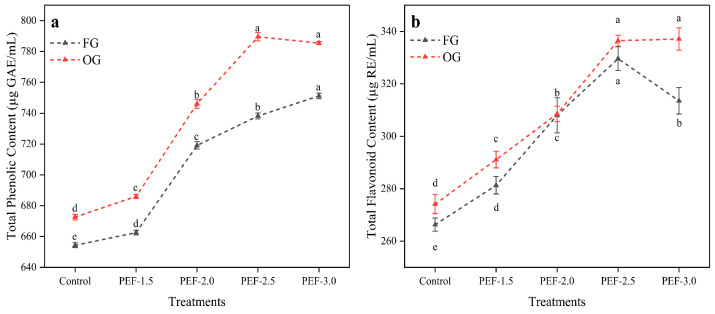
Total phenolic content (**a**) and total flavonoid content (**b**) of fresh and old ginger (juice) treated with PEF. Different letters above the data points indicate statistically significant differences (*p* < 0.05) among treatments.

TPC exhibited a positive correlation with increasing PEF strength, ranging from 654.36 µg GAE/mL in the control group to 751.21 µg GAE/mL at 3.0 kV/cm. The most substantial increase in TPC occurred at 3.0 kV/cm, and significant differences were observed between the treatments (Figure 1a). A similar pattern was observed in OG, where TPC increased from 672.61 µg GAE/mL in the control to 789.57 µg GAE/mL at 2.5 kV/cm. The increased TPC in OG compared with the FG was a result of metabolic changes during maturation, as OG would have accumulated more phenolic compounds in response to environmental pressures during the aging process. The enhancement of phenolic compound extraction by PEF treatment was likely due to the physical disruption of the plant cell structures, which facilitated the release of these bioactive compounds from intracellular compartments. This phenomenon aligns with the findings of previous studies. For example, Medina-Meza, et al. [58] found no adverse effects of PEF on the polyphenol content in raspberry and blueberry purees, suggesting that PEF treatment does not degrade phenolic compounds in all plant matrices. Peiró et al. [59] observed a significant increase in TPC in PEF-treated lemon residues compared with controls, highlighting the effectiveness of PEF in enhancing phenolic extraction. Similarly, Rahaman et al. [55] also reported significant increases in phenolic content in apricot juice samples treated with PEF at 7 and 14 kV/cm, further supporting the notion that PEF can boost phenolic yields in various plant-based products.

Similarly, TFC in FG increased significantly with PEF treatment, from 266.31 µg RE/mL in the control to 329.62 µg RE/mL at 2.5 kV/cm, with the highest increase observed at 2.5 kV/cm. A similar trend was reported by Radnia, et al. [60] in arvaneh plant (mint family), where TFC initially increased with PEF intensity but declined beyond 3.25 kV/cm, which was attributed to the partial degradation of flavonoids under excessive electric field strength. Over-electroporation at high field intensities can induce localized heating, generate strong electric gradients, and promote the formation of reactive oxygen species (ROS). These conditions may lead to the cleavage of chemical bonds in flavonoid molecules or initiate oxidative degradation reactions, ultimately reducing the total flavonoid content. TFC in OG increased from 274.14 µg RE/mL (control) to 337.11 µg RE/mL at 3.0 kV/cm. OG consistently exhibited higher TFC values than FG at all PEF strengths (Figure 1b). Flavonoids, like phenolic compounds, are more effectively released when plant tissues undergo mechanical or electrical treatments such as PEF. The increase in TFC with PEF treatment suggests that the enhanced extraction and solubility of these compounds was due to the disruption of plant cell walls. Similar to TPC, OG displayed higher TFC compared with FG, indicating that older ginger may naturally contain higher flavonoid concentrations. This could be attributed to the accumulation of secondary metabolites as the plant matures [61]. Medina-Meza, et al. [58] observed a 20% increase in flavonoid extraction from raspberry puree due to PEF treatment, while Ahmed, et al. [62] reported an increase in flavonoid content in PEF-treated wheatgrass juice, attributing it to the improved extraction of intracellular components.

The higher TPC and TFC values in OG compared with FG suggested a metabolic shift in older ginger, where there is an increased accumulation of phenolic compounds, and flavonoid content, contributing to its enhanced antioxidant activity. These compounds, particularly tannins, are associated with the plant’s defense mechanisms against environmental pressures, such as physical injury, pathogen invasion, and changes in environmental conditions, like O_2_ and CO_2_ concentrations [63]. As a result, OG exhibited higher levels of TPC, TFC, and condensed tannins compared with FG. PEF treatment enhanced extraction efficiency by disrupting the cellular structure, as demonstrated by SEM analysis, which revealed structural changes that facilitated the release of bioactive compounds.

### 3.4. Total Flavanols and Condensed Tannin Contents of Ginger Juice

**Figure 2:**
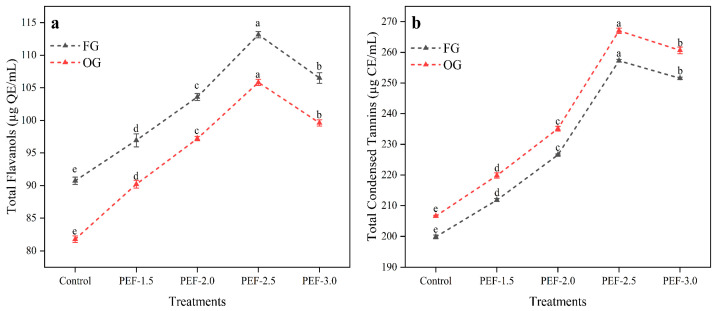
Total flavanols (**a**) and condensed tannin contents (**b**) of fresh and old ginger (juice) treated with PEF. Letters above the data points denote significant differences among treatments (*p* < 0.05).

The total flavanols content in FG increased from 90.73 µg QE/mL (control) to 113.16 µg QE/mL at 2.5 kV/cm, with the highest increase observed at 2.5 kV/cm, followed by a slight decrease at 3.0 kV/cm (106.51 µg QE/mL) (Figure 2a). In OG, the control value was 81.78 µg QE/mL, and the flavanol content increased to 99.62 µg QE/mL at 3.0 kV/cm, with the peak value of 105.83 µg QE/mL observed at 2.5 kV/cm. Plants synthesize bioactive compounds such as flavanols, which play a crucial role in their early defense mechanisms, particularly in response to oxidative stress. These compounds are actively synthesized to protect against environmental stressors like UV radiation, pathogens, and herbivores [64,65]. In contrast, OG undergoes different metabolic pathways during storage, favoring the accumulation of phenolic compounds over flavanols. However, after PEF treatment, the disruption of cell membranes enhances the release of intracellular compounds, facilitating the extraction of flavanols [66,67]. As a result, the flavanol content in OG increased post-PEF, approaching levels found in FG. This suggests that PEF not only improves the extraction efficiency of flavanols but also mobilizes them from the plant’s cellular structures, making them more available, thus reducing the disparity in flavanol content between OG and FG. Flavanols, a subclass of flavonoids, are sensitive to extraction methods, and PEF treatment appears to enhance their release from ginger tissues. The increase in flavanols at moderate PEF strengths (2.0–2.5 kV/cm) suggests that this range optimizes the release of flavanols without excessive degradation or disruption. The slight decrease at 3.0 kV/cm may indicate that intense PEF treatment causes structural damage, reducing flavanol stability or extraction efficiency. Additionally, the higher intensity of the pulsed electric field could generate free radicals, which may contribute to the degradation of flavanols, further compromising the stability and yield of these compounds. Delso, et al. [68] found that juice from grapes treated with PEF showed higher flavanols and non-flavonoid content, with quercetin being the predominant flavanol. The concentration of this flavanol, known for its potent antioxidant properties, was found to be twice as high in PEF-treated grape juice. Grzelka, et al. [69] have also reported that PEF treatment increases flavonoids and flavanols due to the permeabilization of plant cell membranes. Similarly, Yang, et al. [70] have observed an increase in total phenolics and flavonoids in olive oil following PEF treatment.

The CT content in FG increased from 199.86 µg CE/mL (control) to 251.53 µg CE/mL at 3.0 kV/cm, with a peak value of 257.27 µg CE/mL observed at 2.5 kV/cm. In OG, CT increased from 206.59 µg CE/mL (control) to 260.67 µg CE/mL at 3.0 kV/cm, reaching a peak of 267.06 µg CE/mL at 2.5 kV/cm (Figure 2b). The increase in CT in both FG and OG following PEF treatment suggests that PEF enhances the release of these tannins from plant cells, likely through the disruption of cell wall and membrane integrity. The highest increase in CT at 2.5 kV/cm suggests that moderate PEF intensities are most effective for releasing these compounds, while higher intensities may not significantly improve extraction due to potential degradation or destabilization of the compounds. Additionally, OG consistently exhibits higher CT content when compared with FG, which could be attributed to the age-related accumulation of polyphenolic compounds in older ginger. Maza, et al. [71] have reported an increase in tannin extraction in Caladoc and Grenache grapes due to PEF treatment, and Bebek Markovinovic, et al. [72] have observed increased stability in condensed tannins in PEF-treated strawberry juice. Rahmah and Ahsan [73] have also reported enhanced extraction of condensed tannins from Areca seed powder extract following PEF treatment.

PEF treatment significantly influences the total flavanols and CT content in both fresh and old ginger, with moderate pulse strengths (2.0–2.5 kV/cm), proving most beneficial for compound release. At 3.0 kV/cm, although increases in both flavanols and CT were still observed, the rate of increase slowed, indicating the existence of an optimal PEF strength for maximizing bioactive compound extraction. The higher flavanols and CT content in OG compared with FG further supports the notion that aging enhances the concentration of these compounds in ginger.

### 3.5. DPPH^•^ and ABTS^•+^ Inhibition

**Figure 3:**
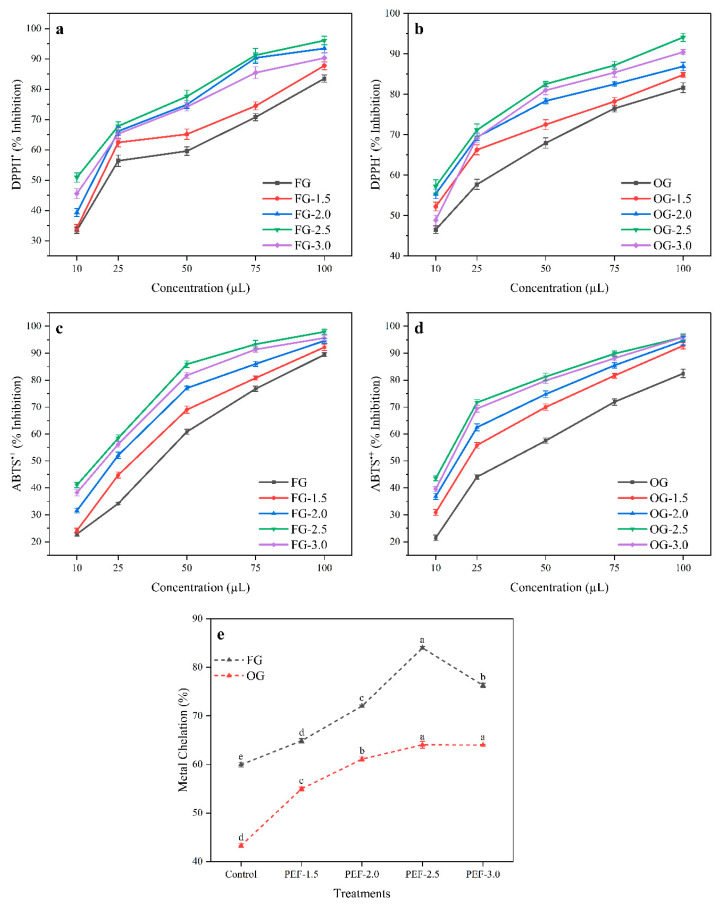
DPPH^•^ inhibition of FG (**a**) and OG (**b**); ABTS^•+^ inhibition of FG (**c**) and OG (**d**); and metal chelation potential (**e**) of FG and OG treated with PEF. Letters above the data points indicate significant variation (*p* < 0.05).

DPPH^•^ and ABTS^•+^ inhibition showed a clear increase with PEF treatment for FG (Figure 3a,c). The baseline DPPH^•^ inhibition in the control sample was 60.75%, and it increased significantly to 76.73% at 2.5 kV/cm, although a slight decrease was observed at 3.0 kV/cm (72.16%). Similarly, the baseline ABTS^•+^ inhibition for FG was 56.82%, which increased progressively to 75.34% at 2.5 kV/cm, before slightly decreasing to 72.64% at the highest PEF intensity (3.0 kV/cm). These results suggest that PEF treatment enhances the antioxidant capacity of FG, but that excessive PEF intensity (3.0 kV/cm) might degrade or alter the bioactive compounds, reducing antioxidant effectiveness. The peak antioxidant activity was consistently observed at 2.5 kV/cm, indicating an optimal PEF intensity for FG.

OG exhibited similar trends in both DPPH^•^ and ABTS^•+^ inhibition. The baseline DPPH^•^ inhibition for OG was 66.05%, which increased to 78.43% at 2.5 kV/cm, but slightly decreased to 74.98% at 3.0 kV/cm (Figure 3b). For ABTS^•+^ inhibition, the baseline was 55.48%, and it peaked at 76.44% at 2.5 kV/cm, before decreasing to 74.56% at 3.0 kV/cm (Figure 3d). OG consistently showed higher antioxidant activity than FG across all PEF intensities. This could be attributed to the higher concentration of bioactive compounds, such as phenols and flavonoids, which are naturally accumulated in older ginger and which contribute to its stronger antioxidant potential. The results suggest that OG benefits from PEF treatment similarly to FG, with the aging process enhancing its bioactive compound content via different metabolic pathways as a result of environmental pressures, thereby boosting its antioxidant capacity and making it more resilient to over-processing.

The increase in antioxidant activity (both DPPH^•^ and ABTS^•+^ inhibition) with PEF treatment suggests that the process improves the bioavailability of antioxidant compounds in ginger juice. Huang, et al. [74] have reported significantly higher DPPH^•^ activity in PEF-treated apricot samples compared with controls. Similarly, Medina-Meza, et al. [58] have demonstrated a significant increase in DPPH^•^ activity in PEF-treated raspberry puree. Regarding ABTS^•+^ inhibition, Yang et al. [70] have observed an increase in both DPPH^•^ and ABTS^•+^ inhibition due to PEF treatment, which aligns with our findings. Moreover, Souli, et al. [75] found an increase in ABTS^•+^ activity in PEF-treated date fruit extracts. These findings highlight that PEF treatment can effectively enhance antioxidant activity, and that the higher levels in OG are likely due to its naturally higher concentrations of phenolic and flavonoid compounds, as demonstrated in TPC and TFC results.

### 3.6. Metal Chelating Potential of Ginger Juice

The metal chelation capacity of FG increased significantly with PEF treatment, rising from 59.94% in the control to 76.29% at 3.0 kV/cm, with the highest increase at 2.5 kV/cm (83.98%). In contrast, OG exhibited a metal chelation capacity increase from 43.29% in the control to 63.97% at 3.0 kV/cm (Figure 3e). While both FG and OG matrixes benefited from PEF treatment, OG showed lower baseline chelation activity compared with FG. However, PEF treatment significantly enhanced the chelation capacity of both samples, suggesting that PEF promotes the release of compounds in ginger capable of binding metal ions, which are beneficial in preventing oxidative stress and metal-catalyzed reactions [76,77]. The higher increase in chelation capacity observed in FG suggests that younger ginger might have a naturally higher potential for metal ion binding.

FG typically contains higher levels of metal-chelating agents such as phenolic acids (e.g., caffeic acid, ferulic acid, and p-coumaric acid). These compounds are essential in the plant’s early defense mechanisms. In contrast, OG tends to follow different metabolic pathways, with leads to higher levels of condensed tannins and phenolic compounds, which are associated with its later-stage defense mechanisms. Although OG may have increased antioxidant activity due to the accumulation of these compounds, FG remains more effective for metal chelation due to its higher concentration of phenolic acids in the stages of growth. The results from this study are consistent with those of the work of Zhang, et al. [78], who reported increased iron chelation in PEF-treated samples, and Wang, et al. [79], who found that PEF treatment significantly increased the chelation ability of melanoidins towards Fe (II). Additionally, these findings are in agreement with Medina-Meza, et al. [58], who observed a significant increase in antioxidant properties in PEF-treated raspberry extracts, supporting the conclusion that PEF is an effective technique for enhancing the bioactivity of ginger products.

### 3.7. SEM and Cell Disintegration Degree of Ginger

**Figure 4:**
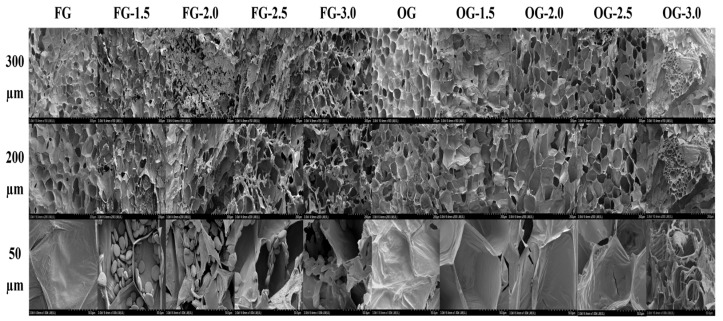
SEM images of FG and OG slices treated with PEF at different intensities.

SEM images revealed the impact of PEF treatment on the microstructure of the FG and OG slices (Figure 4). At low PEF intensity (1.5 kV/cm), the SEM images showed minimal damage, with intact cell walls and smooth surfaces. With increased PEF intensity, there were noticeable disruptions in the cell walls, with ruptures and larger void spaces observed at higher PEF intensities (2.5 and 3.0 kV/cm). The most pronounced microstructural damage was seen at 3.0 kV/cm, where the cell walls were severely disrupted, indicating higher levels of cell disintegration. Similar trends were observed for the OG, with minimal damage at low PEF intensities and greater disruption at higher intensities. The most significant damage in the OG slices occurred at 3.0 kV/cm, where the cell walls appeared severely disrupted, and large areas of cell disintegration were evident. These findings confirmed that the PEF treatment led to cell wall disruption, which increased with higher PEF intensities. The rupture of cell membranes allowed the release of intracellular components, such as bioactive compounds, which would have enhanced the extraction process. The more pronounced microstructural changes in OG compared with FG could be due to the differences in the inherent properties of fresh versus old ginger, such as cell wall rigidity and cellular composition [80,81]. Lohani and Muthukumarappan [82] observed similar results, showing that electroporation increased the porosity of cellular membranes in plant tissues. Faridnia, et al. [83] have also reported significant microstructural differences in potato tissues after PEF treatment, indicating that the PEF’s effect on microstructure varies with electric field strength. Wang, et al. [84] confirmed the electroporation effect of PEF during their study on biomolecule recovery from Chlorella, noting changes in the microstructure.

**Figure 5:**
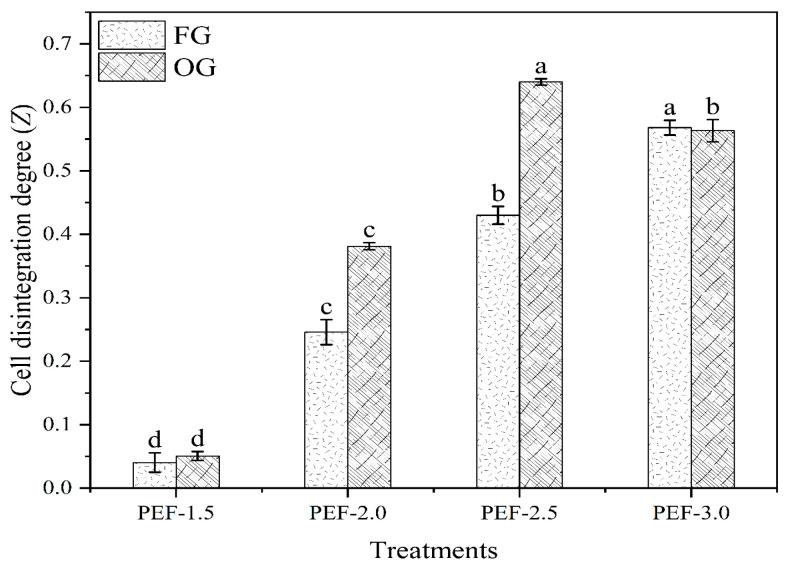
Cell disintegration degree (Z) of FG and OG treated with PEF. Different letters represent significant differences across treatments (*p* < 0.05).

The degree of cell disintegration (*Z*) in FG increased with higher PEF treatment intensities, rising from 0.04 at 1.5 kV/cm to 0.57 at 3.0 kV/cm. The highest disintegration (0.57) occurred at 3.0 kV/cm, indicating more extensive breakdown of cell structures with higher PEF intensities. Similarly, OG showed an increase in cell disintegration degree with higher PEF intensities (Figure 5). The peak disintegration in OG occurred at 2.5 kV/cm (0.64), followed by 3.0 kV/cm (0.56), suggesting that OG reached maximum disintegration at a slightly lower PEF intensity compared with FG. These results correlate with the SEM observations, where higher PEF intensities lead to more severe disruption of cell walls. The differences in cell disintegration between FG and OG may be due to the natural differences in cell structure between the fresh and old ginger, with the OG potentially having softer or more easily disrupted cells. These findings are in line with those of Peiró, Luengo, Segovia, Raso and Almajano [59], who observed a similar trend in the cell disintegration index (Z), noting that the values increased with higher electric field strength and treatment duration, reaching a maximum of 0.55 with the most severe PEF treatment.

The SEM images and cell disintegration degree data demonstrate that PEF treatment significantly disrupts the microstructure of ginger, with 2.5 kV/cm causing more pronounced damage to the cell walls and increasing cell disintegration. This disruption facilitates the release of intracellular bioactive compounds, enhancing their extraction for various applications. OG slices showed a higher degree of disintegration than FG slices, likely due to age-related differences in cell wall strength and composition. Overall, PEF treatment effectively modified the microstructure of the fresh and old ginger, with the degree of disruption increasing with higher PEF intensities, suggesting that PEF could be an effective method for improving the extraction of bioactive compounds from ginger.

### 3.8. Color Parameters of Ginger Juice

PEF treatment significantly affected the optical properties of both fresh and old ginger juice (Table 2). For FG, the L* (lightness) value increased from 17.73 (control) to 28.80 at 2.5 kV/cm, indicating a lightening effect, followed by a slight decrease at 3.0 kV/cm. In OG, the L* value increased from 25.10 (control) to 31.77 at 3.0 kV/cm, suggesting a brightening effect with PEF. The observed increase in L* (lightness) following PEF treatment can be attributed to enhanced cell membrane permeabilization and partial inactivation of oxidative enzymes. PEF induces electroporation of plant cell membranes, facilitating the release of intracellular water, soluble solids, and suspended particulates into the juice matrix. This elevated dispersion of solutes increases turbidity and light scattering, thereby producing a visually lighter appearance and higher L* values [30]. Moreover, PEF is known to inactivate oxidative enzymes such as polyphenol oxidase (PPO), which are primarily responsible for enzymatic browning reactions in plant-based matrices. By reducing PPO activity, PEF limits pigment oxidation and helps maintain a paler color. Supporting this mechanism, previous studies have demonstrated that PEF-treated apple juice exhibited significantly higher lightness when compared with untreated controls, confirming the lightening effect associated with membrane disruption and enzyme inactivation [85]. The a* value (redness/greenness) decreased for both FG and OG, shifting towards more greenness, which likely resulted from the breakdown of chlorophyll. Similarly, the b* value (yellowness/blueness) increased for both types of ginger, indicating a promotion of yellow tones, possibly due to the release of carotenoids (β-carotene and xanthophylls). The total color difference (Δ*E*) reached its highest at 2.5 kV/cm for both FG and OG, suggesting a noticeable change in the overall color. Additionally, saturation (C* values increased, enhancing the vividness and intensity of the juice’s color, while the whiteness index (WI) also increased, further supporting the lightening effect. The increase in non-enzymatic browning (NEB) at 2.5 kV/cm, particularly in both FG and OG, suggests that PEF treatment might induce some Maillard reaction or caramelization of sugars [86]. In OG, the L* value at 2.5 kV/cm (30.77) is actually slightly lower than at 3.0 kV/cm (31.77), despite the overall lightening trend. This suggests that the dark brown melanoidin pigments formed at 2.5 kV/cm partly offset the PEF-induced lightening. Likewise, brown color reflects a mix of green and red components along the a* axis [87]. At 2.5 kV/cm the OG a* value is most negative (strongest green), likely from chlorophyll release, whereas at 3.0 kV/cm a* becomes less negative (more redshifted). The slight redshift of a* at higher field could indicate a tint of red-brown from NEB products. Notably, the b* value jumps substantially at 2.5 kV/cm, consistent with strong yellow-brown pigments. Taken together, the peak Δ*E* at 2.5 kV/cm and these lab shifts imply that NEB and Maillard pigments do produce a darker yellow-brown color in OG, which counteracts the pure lightening effect and is detectable as a plateauing or reduction in L* and a slight a*-axis shift.

The changes in optical properties observed in this study were consistent with those reported in other PEF treatment studies. Carbonell-Capella, et al. [88] reported significantly higher L* values for PEF-treated fruit juices, which they attributed to the modification of juice structure. Similarly, Timmermans, et al. [89] also found increases in L* and b* values for PEF-treated orange juice, suggesting that the treatment enhances brightness and yellowness. They observed a decrease in a* values, which aligns with the findings of this study, and noted that Δ*E* values increased significantly, similar to the results observed here. Yeom, et al. [90] observed a higher hue angle for PEF-treated orange juice compared with heat-pasteurized samples, which suggests a shift towards more yellow-green tones, consistent with the hue shift observed in this study. Furthermore, Delso, et al. [68] reported a significant increase in L*, chroma, and hue angle in PEF-treated red grape juice, supporting the idea that PEF treatment enhances the color quality of fruit juices. These findings suggest that PEF can effectively modify the color of ginger juice, making it more appealing for use in the food and beverage industry.

### 3.9. FT-IR Profile of Ginger Juice

**Figure 6:**
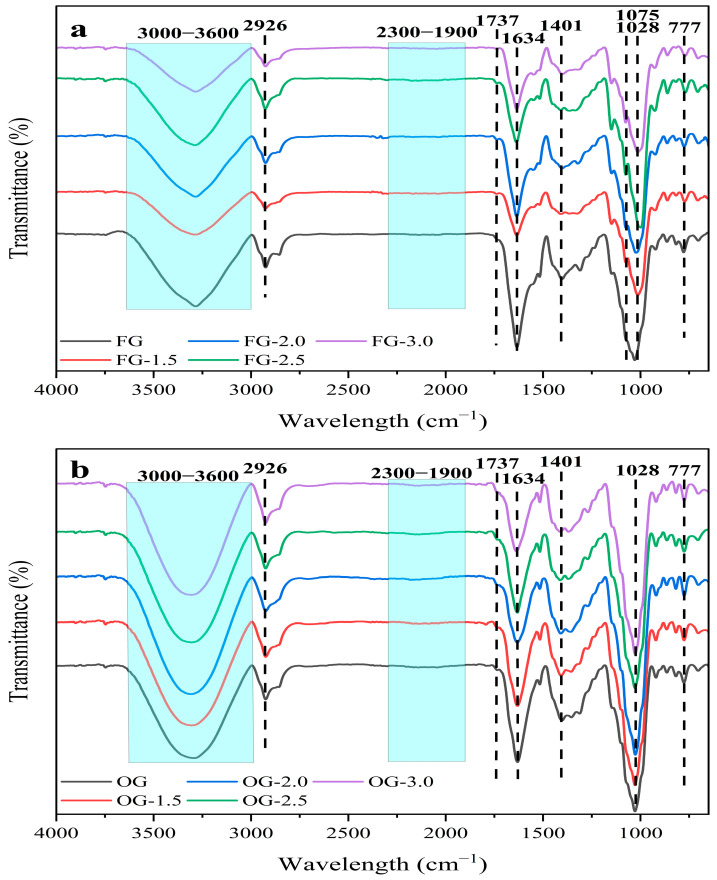
FT-IR spectra of FG (**a**) and OG (**b**) juice treated with PEF.

Table 3 shows the area under the peaks, correlating with the concentration of functional groups associated with those peaks. PEF treatment significantly altered the FT-IR profiles of both FG and OG, particularly at higher treatment intensities, with notable changes observed at 2.5 kV/cm. For FG, the intensity of peaks corresponding to cellulose and phenolic compounds (1028 cm^−1^), water (3600–3000 cm^−1^) [55], and C-H bending vibrations (777 cm^−1^) increased, indicating enhanced extraction and disruption of cellular structures. These changes suggest that PEF treatment facilitates the release of key bioactive compounds, such as cellulose and phenols. For OG, the FT-IR spectra showed even more pronounced changes, with higher peak intensities at 1028 cm^−1^ (cellulose and phenols), 1737 cm^−1^ (aldehydes/ketones), and 2926 cm^−1^ (carboxylic acids) (Figure 6b).

These results indicate that OG contains more complex structures or a higher concentration of bioactive compounds, which were more susceptible to modification and release under PEF treatment. A key observation was the 2926 cm^−1^ peak, which corresponds to C–H stretching vibrations in carboxylic acids [91]. This peak showed an increase in intensity in both FG and OG, particularly at higher PEF intensities. This suggests that PEF treatment may facilitate the release of compounds such as fatty acids or other carboxylated compounds from the ginger matrix, contributing to the enhanced bioactivity of ginger. The increase in the 2926 cm^−1^ peak aligned with the overall trend of increased bioactive compound release with PEF treatment. The peak at 1634 cm^−1^ was associated with the C = C groups in aromatic rings and alkenes [92]. The increase in the 1737 cm^−1^ peak intensity, which corresponds to C = O stretching vibrations in aldehydes and ketones [93], further supports the idea that PEF treatment modifies and enhances the release of a variety of bioactive compounds from both fresh and old ginger.

### 3.10. Volatile Aroma Composition of Ginger Juice

PEF treatment at 2.5 kV/cm had a substantial impact on the volatile compound profiles of both fresh and old ginger (Table 4). Fresh ginger (FG) has the lowest total volatile compound concentration at 88.17 µg/mL, which suggests that the volatile compounds in fresh ginger are less readily extracted due to the intact cellular structure, which acts as a physical barrier, limiting the release of these compounds. After PEF treatment, the total volatile concentration in FG (FG-2.5) increases to 208.98 µg/mL, indicating that PEF treatment enhances the release of volatile compounds by disrupting cell membranes and facilitating their extraction. Key compounds, such as camphene, cyclohexane, and eucalyptol, show increased concentrations after PEF treatment, contributing to the more intense aroma and flavor. In contrast, old ginger (OG) naturally contains a higher concentration of volatile compounds (392.73 µg/mL), likely due to the aging process, which leads to the accumulation of certain volatiles through chemical transformations or breakdowns in the ginger root. Prominent compounds in OG juice include caryophyllene, α-farnesene, neral, and β-bisabolene, which contribute to its richer and more complex flavor profile. PEF treatment on old ginger (OG-2.5) resulted in a dramatic increase in the total volatile compound concentration to 1091.23 µg/mL, reflecting the effectiveness of PEF in breaking down the more rigid and less permeable cell wall structure of aged ginger, as, over time, the cell walls become more lignified or contain more complex polysaccharides, leading to a firmer and less flexible structure, in turn enabling the release of a greater quantity of volatile compounds. PEF treatment enhances the release of compounds like neral and α-farnesene, which were previously less accessible.

Regarding the number of volatile compounds, fresh ginger (FG) contains 26 compounds, while PEF-treated fresh ginger (FG-2.5) showed an increase to 39 compounds, highlighting that PEF treatment also helps release a broader range of volatile compounds. On the other hand, old ginger (OG) contained only 28 compounds, indicating that aging can reduce the diversity of volatile compounds. However, after PEF treatment, the number of compounds in old ginger increased to 35, showing that PEF treatment not only increases the concentration of existing volatile compounds (camphene concentration increased from 17.76 to 37.67 µg/mL, while the concentration of copaene increased from 3.06 to 10.48 µg/mL) but also helps release additional volatiles that were less accessible in untreated old ginger (carotol, epi-Bicyclosesquiphellandrene, and γ-muurolene). The results demonstrate the potential of PEF as an efficient method for improving the flavor, aroma, and medicinal properties of ginger, especially in aged ginger, which tends to have a more complex and resistant cellular structure. These findings are particularly relevant for applications in the food, flavor, and pharmaceutical industries, where the quality of volatile compounds plays a crucial role in the sensory and therapeutic qualities of ginger-based products. Lee, et al. [94] have observed an enhanced retention of flavor compounds in PEF-treated orange juices as compared with control. Timmermans, et al. [89] have observed a slight rise in α-terpinene, 4-terpineol, and α-terpineol due to PEF, as compared with the quantities in untreated orange juice.

### 3.11. Gingerols and Shogaols Composition of Ginger Juice

**Figure 7:**
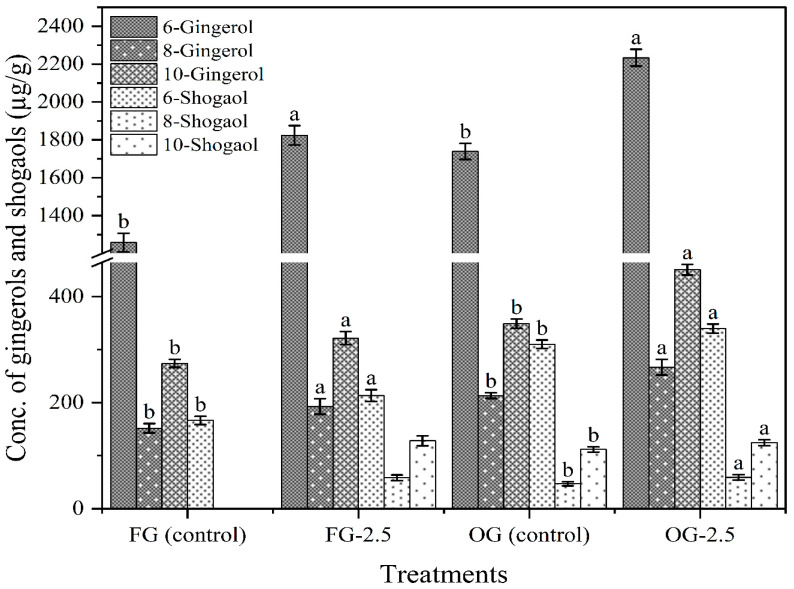
6-, 8-, 10-Gingerol and 6-, 8-, 10-shogoal contents in FG and OG treated with PEF (2.5 kV/cm). Different letters indicate statistically significant differences (*p* < 0.05) among treatments.

The results demonstrate that PEF treatment at 2.5 kV/cm significantly enhances the levels of both gingerols and shogaols in both fresh and old ginger juice (Figure 7). In FG, the baseline levels of gingerols 6-, 8-, and 10-gingerol were 1257.88, 151.87, and 274.21 µg/g, respectively. After PEF treatment (FG-2.5), these values increased substantially to 1824.05 (45.01% increase), 192.96 (27.06% increase), and 321.77 µg/g (17.34% increase), indicating a marked increase in gingerol content. Similarly, the shogaol content in fresh ginger also increased, with 6-shogaol rising from 166.43 in control (FG) to 213.52 µg/g (28.29% increase) after PEF treatment. The 8-shogaol and 10-shogaol, were not detectable in the control (FG) and measured at 58.36 and 128.15 µg/g in the PEF-treated samples respectively, further emphasizing the enhancement of bioactive compounds through PEF treatment. This indicates that PEF treatment is effective in promoting the conversion of gingerols to shogaols, which are known to have higher bioactivity and potential health benefits, though this phenomenon has not been extensively studied yet.

In the case of old ginger (OG), baseline levels were higher, with gingerol content for 6-, 8-, and 10-gingerol being 1739.16, 213.35, and 348.95 µg/g, respectively. PEF treatment (OG-2.5) led to significant increases in these compounds, reaching 2233.60 (28.43% increase), 267.04 (25.17% increase), and 450.90 µg/g (29.22% increase), respectively. Similarly, shogaol content in OG saw a significant increase, with 6-shogaol rising from 310.31 to 339.63 µg/g (9.45% increase), 8-shogaol from 47.06 to 59.09 µg/g (25.56% increase), and 10-shogaol from 111.76 to 124.41 µg/g (11.32% increase). The higher concentrations in OG compared with FG suggest that age-related differences in ginger composition may influence the levels of these bioactive compounds.

This increase in gingerols (6-, 8-, and 10-gingerol) can be related to the increased total phenolics observed in this study as these are major phenolics found in ginger [95]. Ghosh, et al. [96] have also reported a positive correlation between 6-gingerol and total phenolic content (TPC). These results suggest that PEF treatment not only increases the levels of key bioactive compounds but may also enhance the extraction efficiency of these compounds in both fresh and old ginger, regardless of their initial concentration. The increase in gingerols and shogaols in both ginger types highlights the potential of PEF as an effective, non-thermal processing technique for enhancing the nutritional and phytochemical quality of ginger, which could lead to improved bioactive properties in ginger juice and other ginger-based products.

## 4. Conclusions

This study provides new insights into how tissue maturity modulates plant responses to non-thermal processing, specifically pulsed electric field (PEF) treatment. By systematically comparing fresh (young) and old (mature) ginger tissues, we demonstrated that maturity-associated differences in cellular architecture, electrolyte composition, and lignification significantly influence the dielectric breakdown behavior and subsequent extraction efficiency. The findings reveal that mature plant tissues, due to their enhanced dielectric properties and structural rigidity, respond more favorably to PEF, facilitating superior cell disintegration, compound release, and aroma preservation. Moreover, the integration of SEM, FT-IR, HPLC, and volatile profiling techniques confirmed that PEF-induced electro-permeabilization operates through a tissue-dependent mechanism that governs both biochemical and structural transformations. These insights advance our understanding of plant tissue engineering under non-thermal technologies and suggest that targeted application of PEF based on tissue maturity can optimize the functional, nutritional, and sensory quality of plant-based products. This mechanistic understanding paves the way for precision processing strategies in the food, pharmaceutical, and cosmetic industries.

## Figures and Tables

**Table 1:** Effects of PEF treatment on the physicochemical properties of fresh and old ginger.

Sample	Temp. (°C)	pH	EC (μS/cm)	°Brix (%)	Yield (%)	Cloud Value	Cloud Stability
FG	24.17 ± 0.61 ^e^	7.23 ± 0.02 ^a^	223.37 ± 3.96 ^e^	2.33 ± 0.11 ^c^	69.94 ± 1.21 ^d^	2.46 ± 0.008 ^d^	71.98 ± 0.32 ^d^
FG-1.5	26.35 ± 0.54 ^d^	7.09 ± 0.05 ^b^	238.00 ± 2.45 ^d^	2.83 ± 0.09 ^b^	76.38 ± 1.07 ^c^	2.53 ± 0.006 ^c^	88.79 ± 0.49 ^b^
FG-2.0	29.19 ± 0.28 ^c^	7.02 ± 0.03 ^bc^	297.33 ± 4.11 ^c^	3.07 ± 0.09 ^ab^	79.77 ± 1.22 ^b^	2.56 ± 0.011 ^b^	91.84 ± 0.76 ^a^
FG-2.5	37.83 ± 0.59 ^b^	6.99 ± 0.02 ^c^	348.84 ± 3.44 ^b^	3.08 ± 0.05 ^ab^	84.16 ± 1.13 ^a^	2.59 ± 0.009 ^a^	92.38 ± 0.57 ^a^
FG-3.0	43.39 ± 0.31 ^a^	6.98 ± 0.04 ^c^	390.33 ± 2.49 ^a^	3.20 ± 0.08 ^a^	80.57 ± 0.90 ^b^	2.57 ± 0.008 ^ab^	86.68 ± 0.79 ^c^
Results are shown as mean values ± SD in triplicate. Values in the same columns showing the letters differ significantly (*p* < 0.05).							
OG	22.97 ± 0.29 ^e^	6.51 ± 0.02 ^a^	254.0 ± 2.64 ^e^	3.25 ± 0.07 ^c^	81.93 ± 0.24 ^d^	2.73 ± 0.008 ^d^	82.83 ± 0.34 ^d^
OG-1.5	25.96 ± 0.29 ^d^	6.45 ± 0.01 ^b^	258.3 ± 1.70 ^d^	4.30 ± 0.16 ^b^	85.21 ± 1.31 ^c^	2.85 ± 0.005 ^b^	89.40 ± 0.31 ^c^
OG-2.0	30.89 ± 0.47 ^c^	6.41 ± 0.02 ^c^	332.0 ± 3.27 ^c^	4.47 ± 0.12 ^ab^	87.58 ± 0.73 ^b^	2.85 ± 0.002 ^b^	91.25 ± 0.19 ^b^
OG-2.5	36.43 ± 0.24 ^b^	6.34 ± 0.02 ^d^	389.7 ± 2.37 ^a^	4.56 ± 0.06 ^ab^	90.85 ± 0.68 ^a^	2.86 ± 0.003 ^a^	91.96 ± 0.65 ^a^
OG-3.0	41.99 ± 0.25 ^a^	6.30 ± 0.02 ^d^	372.7 ± 2.49 ^b^	4.60 ± 0.08 ^a^	89.19 ± 0.39 ^ab^	2.86 ± 0.001 ^ab^	90.48 ± 0.34 ^b^
Values are means ± standard deviations. Values within each column with different letters are significantly different (*p* < 0.05).							

**Table 2:** Colorimetric analysis of fresh and old ginger juice samples treated with PEF.

Sample	L*	a*	b*	Δ*E*	Hue (hº)	C*	WI	NEB
FG	17.73 ± 0.74 ^c^	0.20 ± 0.08 ^a^	11.93 ± 0.29 ^d^	-	89.04 ± 0.39 ^d^	11.94 ± 0.29 ^d^	16.87 ± 0.74 ^d^	0.243 ± 0.003 ^e^
FG-1.5	27.27 ± 0.24 ^b^	−0.33 ± 0.12 ^b^	14.67 ± 0.05 ^b^	10.01 ± 0.87 ^b^	91.30 ± 0.48 ^c^	14.67 ± 0.05 ^b^	25.80 ± 0.23 ^bc^	0.257 ± 0.003 ^d^
FG-2.0	28.47 ± 0.47 ^a^	−0.57 ± 0.05 ^c^	16.13 ± 0.37 ^a^	11.63 ± 0.32 ^a^	92.01 ± 0.17 ^c^	16.14 ± 0.37 ^a^	26.67 ± 0.40 ^ab^	0.301 ± 0.004 ^c^
FG-2.5	28.80 ± 0.29 ^a^	−1.00 ± 0.05 ^d^	15.47 ± 0.29 ^a^	11.69 ± 0.37 ^a^	93.70 ± 0.35 ^b^	15.50 ± 0.28 ^a^	27.13 ± 0.25 ^a^	0.331 ± 0.003 ^a^
FG-3.0	26.73 ± 0.34 ^b^	−1.23 ± 0.09 ^e^	13.47 ± 0.41 ^c^	9.31 ± 0.65 ^b^	95.25 ± 0.54 ^a^	13.52 ± 0.40 ^c^	25.49 ± 0.26 ^c^	0.315 ± 0.005 ^b^
Values are means ± standard deviations. Values within each column with different letters are significantly different (*p* < 0.05).								
OG	25.10 ± 0.54 ^c^	−0.90 ± 0.08 ^a^	6.00 ± 0.29 ^e^	-	98.51 ± 0.37 ^b^	6.07 ± 0.30 ^e^	24.85 ± 0.52 ^d^	0.281 ± 0.003 ^e^
OG-1.5	28.6 ± 0.22 ^b^	−1.70 ± 0.08 ^b^	9.20 ± 0.14 ^d^	4.92 ± 0.51 ^c^	100.46 ± 0.37 ^ab^	9.36 ± 0.15 ^d^	27.99 ± 0.20 ^bc^	0.296 ± 0.001 ^d^
OG-2.0	28.63 ± 0.5 ^b^	−2.20 ± 0.14 ^c^	10.90 ± 0.08 ^c^	6.29 ± 0.63 ^c^	101.41 ± 0.72 ^a^	11.12 ± 0.08 ^c^	27.77 ± 0.49 ^c^	0.315 ± 0.003 ^c^
OG-2.5	30.77 ± 0.33 ^ab^	−2.73 ± 0.12 ^e^	15.20 ± 0.29 ^a^	10.97 ± 0.67 ^a^	100.21 ± 0.65 ^ab^	15.44 ± 0.27 ^a^	29.06 ± 0.27 ^b^	0.347 ± 0.005 ^a^
OG-3.0	31.77 ± 0.26 ^a^	−2.50 ± 0.08 ^d^	12.03 ± 0.42 ^b^	9.24 ± 0.95 ^b^	101.76 ± 0.76 ^a^	12.29 ± 0.39 ^b^	30.67 ± 0.26 ^a^	0.335 ± 0.002 ^b^
Results are shown as mean values ± SD in triplicate. Values in the same columns showing the letters differ significantly (*p* < 0.05).								

**Table 3:** FT-IR peak analysis of fresh and old ginger juice samples treated with PEF.

No.	Peak	FG	FG-1.5	FG-2.0	FG-2.5	FG-3.0	OG	OG-1.5	OG-2.0	OG-2.5	OG-3.0	Compounds
		Area										
1	777	186.7	87.6	108.0	113.7	81.7	174.4	232.4	134.5	183.9	131.2	C–H bending out-of-plane vibrations
2	1028	2336.0	2820.8	3274.0	3951.7	2912.9	5195.4	5995.3	6438.8	6587.7	6130.8	Cellulose and phenols
3	1075	1006.2	1205.4	1609.6	1711.8	1317.6	-	-	-	-	-	Cellulose and phenols
4	1401	1423.2	1289.5	1790.0	1812.2	1361.8	2753.3	2698.5	2951.0	2549.1	2409.7	CH_2_ or CH_3_ bending vibrations, indicating aliphatic chains
5	1634	1805.9	1320.9	2622.8	1926.9	1982.1	2839.9	2493.5	2045.8	2342.0	2099.9	C = C stretching in aromatic rings or amide (Terpenes like zingiberene, camphene, β-elemene, limonene)
6	1737	197.7	201.2	88.8	64.5	37.5	57.6	78.7	74.9	88.8	68.8	C = O stretching vibrations (aldehydes and ketones)
7	2300–1900	125.9	31.7	36.6	54.1	32.2	298.9	303.2	360.2	304.2	392.6	Aromatic combination and overtone bands
8	2926	642.2	661.8	671.1	859.6	763.8	1023.2	1226.7	1094.9	1233.2	1311.2	C–H stretching vibrations (carboxylic acids)
9	3600–3000	7177.9	4238.6	3900.2	6479.6	4246.7	9810.2	10,877.6	12,221.3	11,622.0	11,627.9	O–H stretching vibrations (water)

**Table 4:** Effect of PEF treatment on the volatile profiles of fresh and old ginger juice.

Sr. No.	RT	Compound	Type	Match Factor	Formula	CAS	Concentration of Compound (µg/mL)			
							FG	FG-2.5	OG	OG-2.5
1	9.19	(1R)-2,6,6-Trimethylbicyclo [3.1.1]hept-2-ene	Monoterpene	98.0	C_10_H_16_	7785-70-8	2.03	3.85	5.92	13.29
2	10.58	Camphene	Monoterpene	98.2	C_10_H_16_	79-92-5	6.49	13.07	17.76	37.67
3	13.53	Cyclopentasiloxane, decamethyl-	Organosilicon compound	97.9	C_10_H_30_O_5_Si_5_	541-02-6	1.43	3.13	5.59	7.29
4	14.11	Bicyclo [3.1.1]heptane, 6,6-dimethyl-2-methylene-, (1S)-	Monoterpene	97.1	C_10_H_16_	18172-67-3	1.05	0.68	2.96	2.66
5	14.20	α-Phellandrene	Monoterpene	94.5	C_10_H_16_	99-83-2	0.34	0.55	8.52	17.14
6	15.57	D-Limonene	Monoterpene	98.5	C_10_H_16_	5989-27-5	1.41	2.66	3.42	7.70
7	15.94	Eucalyptol	Ether	97.6	C_10_H_18_O	470-82-6	11.21	22.62	47.11	42.24
8	15.99	β-Phellandrene	Monoterpene	93.7	C_10_H_16_	555-10-2		7.09		
9	19.16	1,3-Cyclohexadiene, 1-methyl-4-(1-methylethyl)-	Monoterpene	95.3	C_10_H_16_	99-86-5		0.63		
10	20.49	2-Heptanol	Alcohol	93.8	C_7_H_16_O	543-49-7	0.46	1.03	1.51	
11	20.96	Cyclohexasiloxane, dodecamethyl-	Organosilicon compound	94.9	C_12_H_36_O_6_Si_6_	540-97-6	0.59	1.49	2.95	4.19
12	21.56	5-Hepten-2-one, 6-methyl-	Ketone	96.4	C_8_H_14_O	110-93-0	0.64	1.38	4.81	
13	27.66	1,2,4-Metheno-1H-indene, octahydro-1,7a-dimethyl-5-(1-methylethyl)-, [1S-(1α,2α,3aβ,4α,5α,7aβ,8S*)]-	Sesquiterpene	97.2	C_15_H_24_	22469-52-9	0.42	0.54	1.65	5.48
14	28.07	Copaene	Sesquiterpene	96.9	C_15_H_24_	3856-25-5	0.70	0.78	3.06	10.48
15	29.30	(+)-2-Bornanone	Ketone	96.3	C_10_H_16_O	464-49-3		0.63		
16	30.23	(+)-3-Carene	Monoterpene	96.0	C_10_H_16_	498-15-7	1.22	2.31	3.90	3.39
17	31.78	Bicyclo [2.2.1]heptan-2-ol, 1,7,7-trimethyl-, acetate, (1S-endo)-	Ester	97.3	C_12_H_20_O_2_	5655-61-8		0.72		
18	32.11	Cyclohexane, 1-ethenyl-1-methyl-2,4-bis(1-methylethenyl)-, [1S-(1α,2β,4β)]-	Sesquiterpene	96.7	C_15_H_24_	515-13-9	0.97	1.20	3.05	8.65
19	32.39	2-Undecanone	Ketone	95.7	C_11_H_22_O	112-12-9	0.49	0.75		
20	32.61	4-Terpinenyl acetate	Ester	94.0	C_12_H_20_O_2_	4821-04-9	0.44	0.89	1.70	
21	34.36	Aromandendrene	Sesquiterpene	93.8	C_15_H_24_	489-39-4		0.51		
22	34.36	Alloaromadendrene	Sesquiterpene	96.7	C_15_H_24_	25246-27-9			2.06	6.96
23	34.99	1H-Cycloprop[e]azulene, decahydro-1,1,7-trimethyl-4-methylene-	Sesquiterpene	85.0	C_15_H_24_	72747-25-2		0.35		
24	34.99	Caryophyllene	Sesquiterpene	94.0	C_15_H_24_	87-44-5			1.79	5.96
25	35.51	Naphthalene, decahydro-4a-methyl-1-methylene-7-(1-methylethylidene)-, (4aR-trans)-	Sesquiterpene	95.6	C_15_H_24_	515-17-3				3.04
26	35.81	Carveol	Alcohol	95.8	C_10_H_16_O	99-48-9	19.29	23.39	50.90	46.90
27	36.02	α-Cuprenene	Sesquiterpene	89.2	C_15_H_24_	29621-78-1		1.15		
28	36.04	.gamma.-Muurolene	Sesquiterpene	92.1	C_15_H_24_	30021-74-0				8.66
29	36.28	α-Terpineol	Alcohol	95.2	C_10_H_18_O	98-55-5	2.63	5.93	9.68	7.45
30	36.53	endo-Borneol	Alcohol	96.0	C_10_H_18_O	507-70-0		3.75	6.40	5.03
31	36.92	(+)-epi-Bicyclosesquiphellandrene	Sesquiterpene	92.8	C_15_H_24_	54274-73-6		2.63		25.52
32	37.28	(3R,3aS,8aS)-3,6,8,8-Tetramethyl-2,3,4,7,8,8a-hexahydro-1H-3a,7-methanoazulene	Sesquiterpene	93.8	C_15_H_24_	22567-43-7		31.05		3.60
33	37.38	10s,11s-Himachala-3(12),4-diene	Sesquiterpene	85.2	C_15_H_24_	60909-28-6				227.72
34	37.60	β-Bisabolene	Sesquiterpene	96.4	C_15_H_24_	495-61-4	5.95	5.38	27.95	71.47
35	37.74	β-Panasinsene	Sesquiterpene	95.3	C_15_H_24_	1000159-39-0	1.75	1.79	5.52	
36	37.74	1H-Cyclopropa[a]naphthalene, decahydro-1,1,3a-trimethyl-7-methylene-, [1aS-(1aα,3aα,7aβ,7bα)]-	Sesquiterpene	94.0	C_15_H_24_	20071-49-2				16.20
37	37.96	Neral	Aldehyde	91.8	C_10_H_16_O	106-26-3		32.60	81.83	115.18
38	38.43	α-Farnesene	Sesquiterpene	94.3	C_15_H_24_	502-61-4	7.51	7.85	43.73	101.08
39	38.86	Bicyclo [3.1.0]hex-2-ene, 4-methyl-1-(1-methylethyl)-	Monoterpene	85.2	C_10_H_16_	28634-89-1			0.96	
40	39.17	Citronellol	Alcohol	93.6	C_10_H_20_O	106-22-9	0.94	1.64	2.48	
41	39.17	Carotol	Alcohol	85.9	C_15_H_26_O	465-28-1		0.62		3.11
42	39.37	(2S,4aR,8aR)-4a,8-Dimethyl-2-(prop-1-en-2-yl)-1,2,3,4,4a,5,6,8a-octahydronaphthalene	Sesquiterpene	93.0	C_15_H_24_	123123-37-5				2.20
43	39.40	(-)-α-Panasinsen	Sesquiterpene	94.4	C_15_H_24_	56633-28-4				5.57
44	39.57	1H-3a,7-Methanoazulene, octahydro-3,8,8-trimethyl-6-methylene-, [3R-(3α,3aβ,7β,8aα)]-	Sesquiterpene	94.9	C_15_H_24_	546-28-1	11.89	10.15		4.77
45	39.61	Cyclohexene, 3-(1,5-dimethyl-4-hexenyl)-6-methylene-, [S-(R*,S*)]-	Sesquiterpene	96.6	C_15_H_24_	20307-83-9				136.93
46	39.82	Benzene, 1-(1,5-dimethyl-4-hexenyl)-4-methyl-	Sesquiterpene	98.1	C_15_H_22_	644-30-4	6.45	7.37	41.96	120.63
47	42.72	1,5-Cyclodecadiene, 1,5-dimethyl-8-(1-methylethylidene)-, (E,E)-	Sesquiterpene	95.4	C_15_H_24_	15423-57-1	0.61	0.69		5.31
48	42.73	Azulene, 1,2,3,3a,4,5,6,7-octahydro-1,4-dimethyl-7-(1-methylethenyl)-, [1R-(1α,3aβ,4α,7β)]-	Monoterpene	94.1	C_15_H_24_	22567-17-5				2.36
49	43.46	(+)-3-Carene	Sesquiterpene	95.3	C_10_H_16_	498-15-7	1.26	5.70	3.59	5.37
50	49.80	(8R,8aS)-8,8a-Dimethyl-2-(propan-2-ylidene)-1,2,3,7,8,8a-hexahydronaphthalene	Sesquiterpene	89.0	C_15_H_22_	27840-40-0		0.39		
			Total volatile compounds concentration				88.17	208.98	392.73	1091.23
			No. of volatile compounds				26	39	28	35

## Data Availability

The original contributions presented in this study are included in the article. Further inquiries can be directed to the corresponding authors.
